# Supplementary material for: Stability of Non-Ionic Surfactant Vesicles Loaded with Rifamycin S
Source: Pharmaceutics. 2022 Nov 28;14(12):2626. doi: 10.3390/pharmaceutics14122626 (PMC9785864; doi:10.3390/pharmaceutics14122626)
Supplement: Supplementary file 1 [file pharmaceutics-14-02626-s001.zip › pharmaceutics-2029208-supplementary.pdf]

Stability of non-ionic surfactant vesicles loaded with Rifamycin S

V. Marchianò [1,2], M. Matos[1], I. Marcet[1], M.P. Cabal [3], G. Gutiérrez[1], M.C. Blanco-López[2*]

[1] Department of Chemical and Environmental Engineering, University of Oviedo, Julián Clavería 8, 33006 Oviedo, Spain.
[2]Department of Physical and Analytical Chemistry, University of Oviedo, Julián Clavería 8, 33006 Oviedo, Spain
[3] Departament of Organic and Inorganic Chemistry, Instituto de Química Organometálica "Enrique Moles", Universidad de Oviedo c/ Julián Clavería 8, 33006 Oviedo, Spain
* Correspondence: cblanco@uniovi.es; gutierrezgemma@uniovi.es

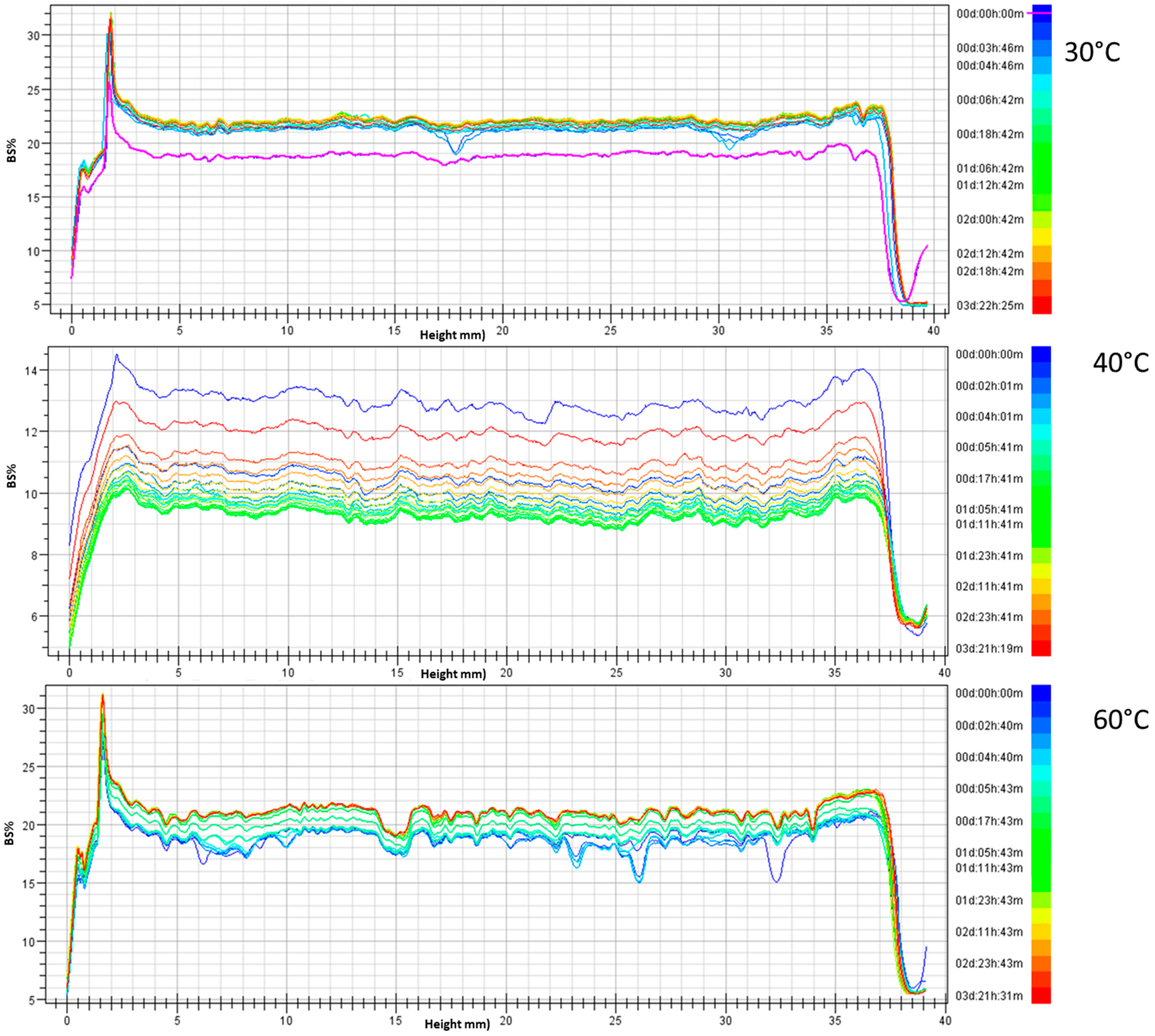

Figure S1. Graph of stability analysis for 3 days obtained from Turbiscan Lab Expert (Formulaction Co., France) of niosomes with Rifamicyn S in MilliQ water at 3 temperatures: 30°C, 40°C and 60°C

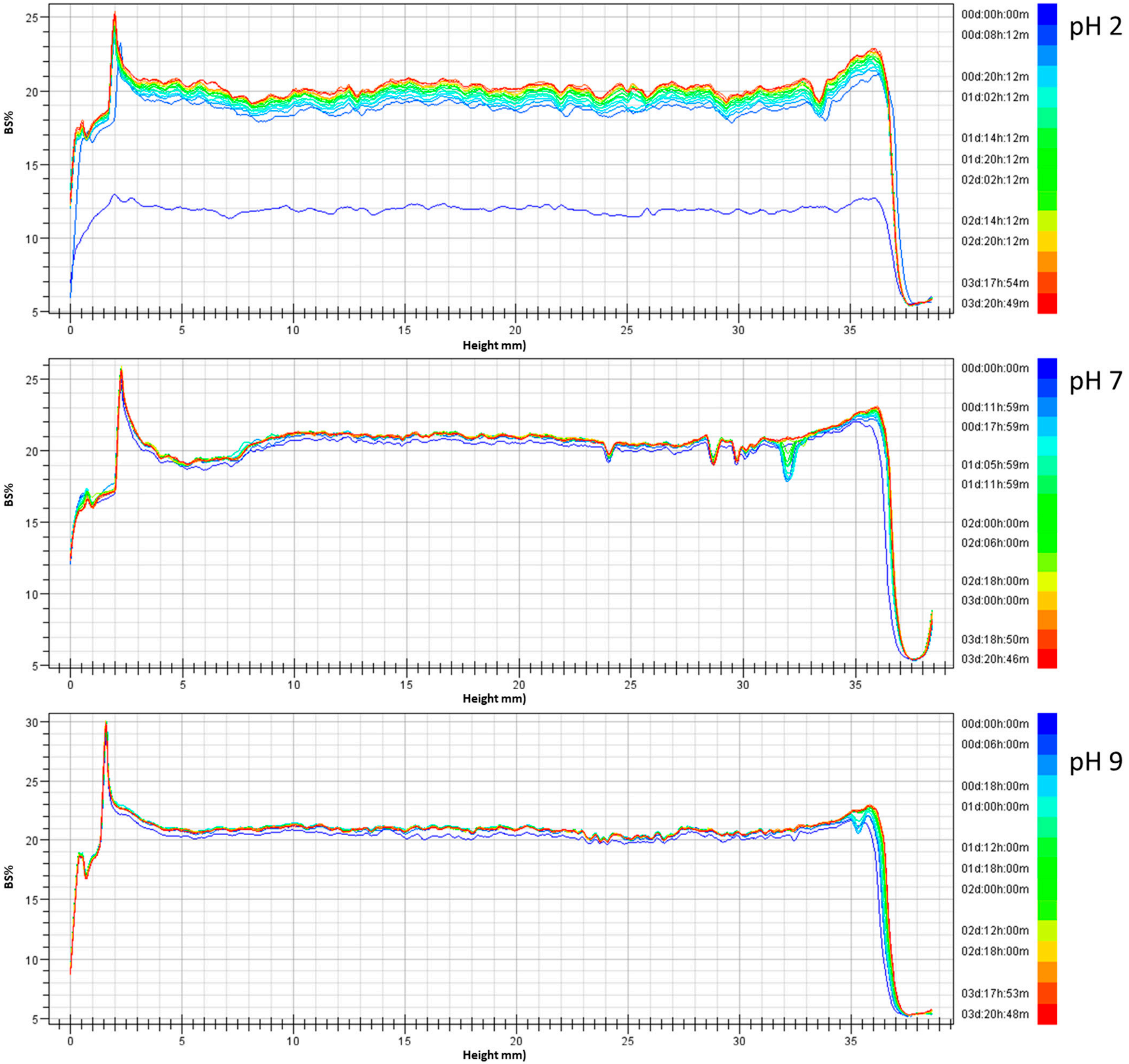

Figure S2. Graph of stability analysis for 3 days obtained from Turbiscan Lab Expert (Formulaction Co., France) of niosomes with Rifamicyn S in MilliQ water at 3 pH: 2, 7 and 9

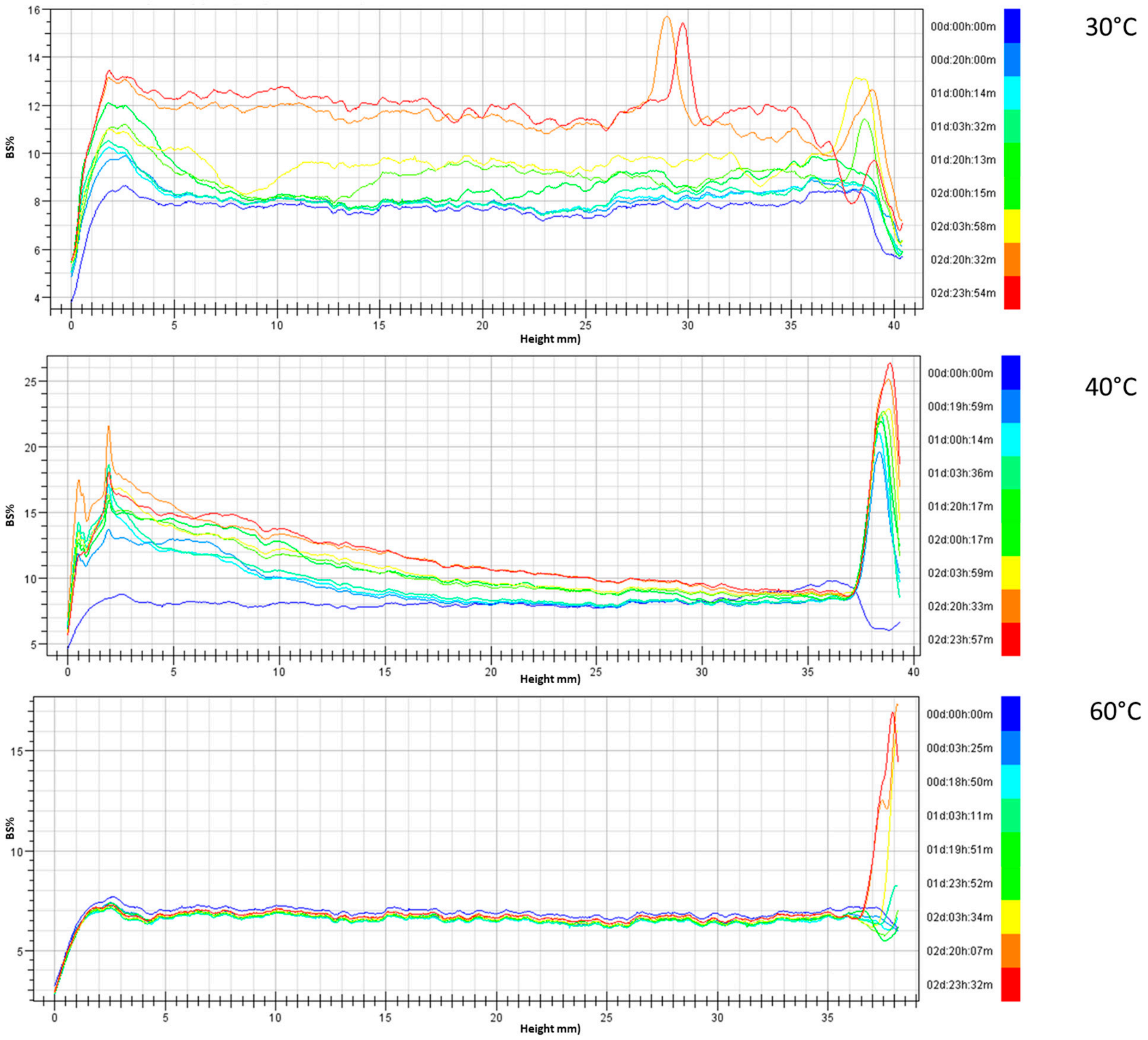

Figure S3. Graph of stability analysis for 3 days obtained from Turbiscan Lab Expert (Formulaction Co., France) of niosomes with Rifamicyn S in MilliQ water and glycerol (60:40) at 3 temperatures: 30°C, 40°C and 60°C

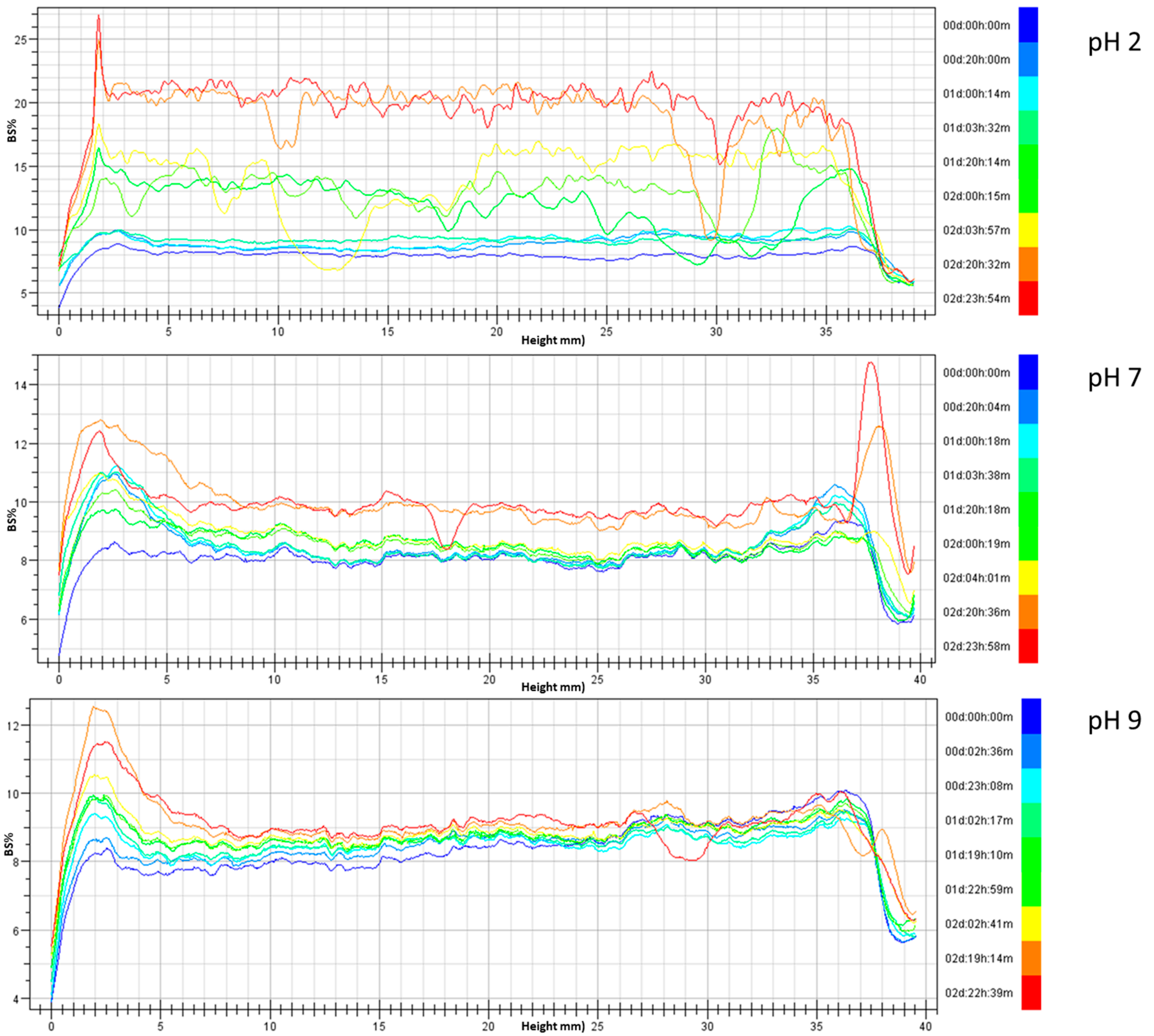

Figure S4. Graph of stability analysis for 3 days obtained from Turbiscan Lab Expert (Formulaction Co., France) of niosomes with Rifamicyn S in MilliQ water and glycerol (60:40) at 3 pH: 2, 7 and 9

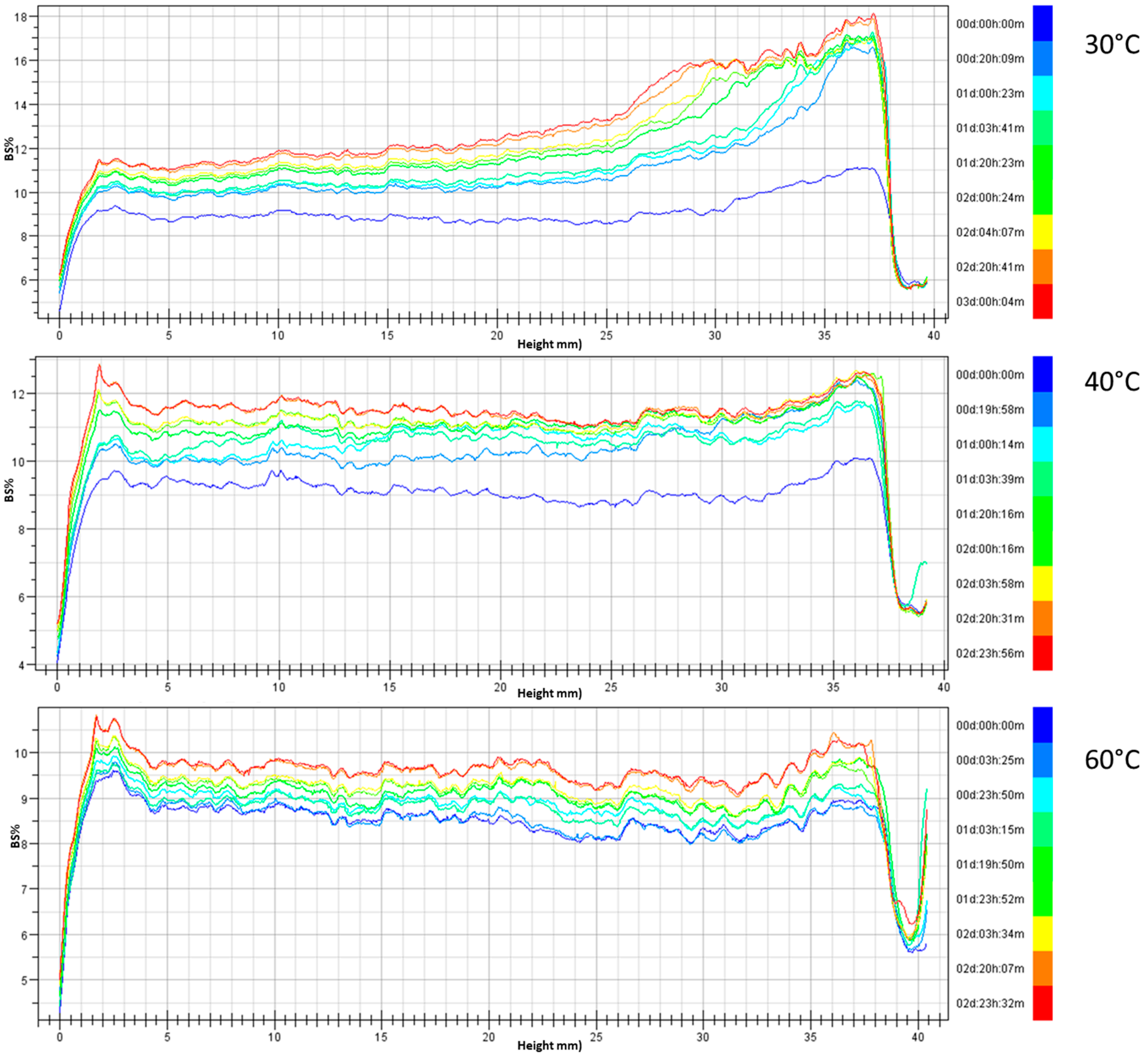

Figure S5. Graph of stability analysis for 3 days obtained from Turbiscan Lab Expert (Formulaction Co., France) of niosomes with Rifamicyn S in MilliQ water andPEG400 (55,7:44,3) at 3 temperatures: 30°C, 40°C and 60°C

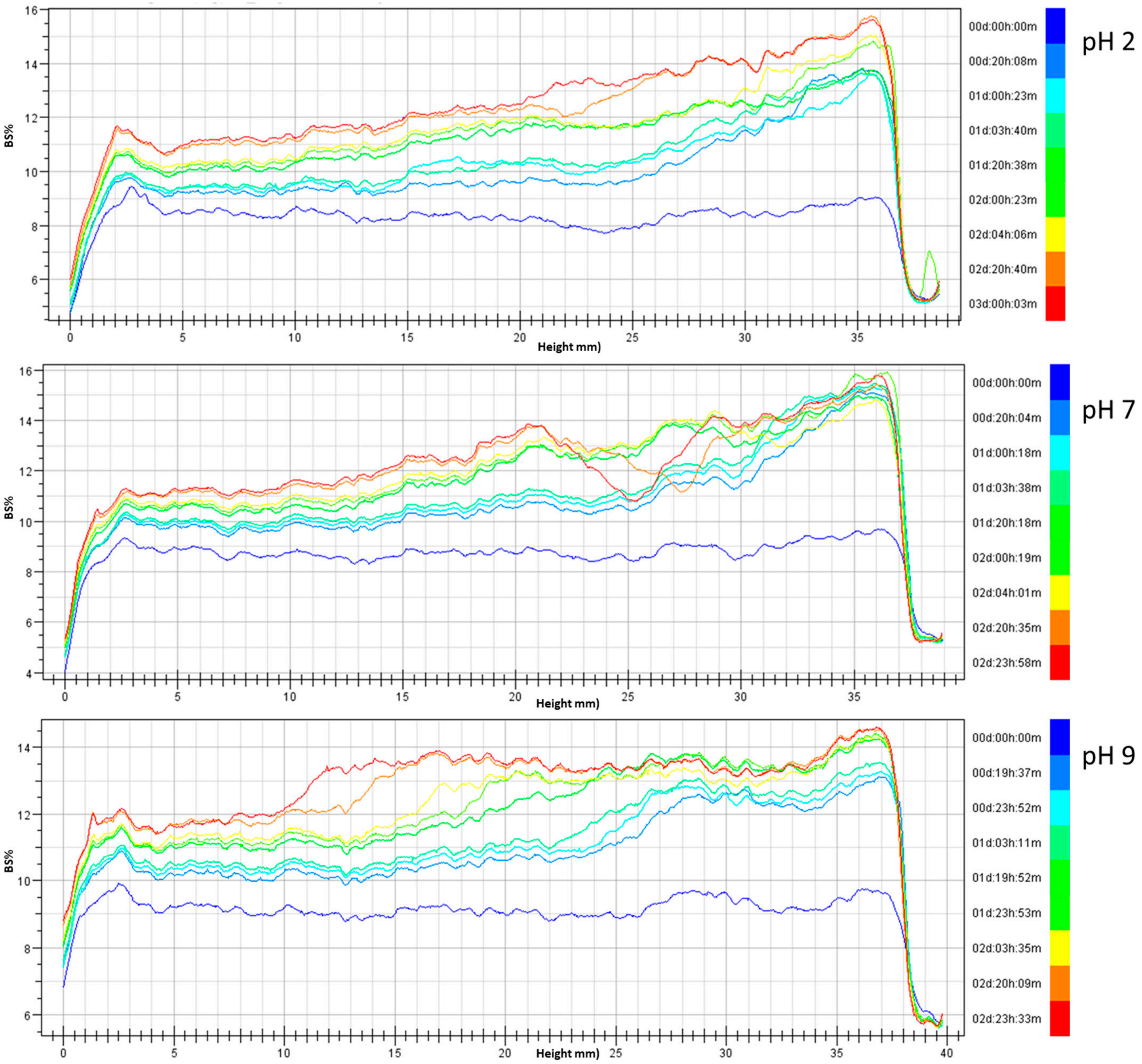

Figure S6. Graph of stability analysis for 3 days obtained from Turbiscan Lab Expert (Formulaction Co., France) of niosomes with Rifamicyn S in MilliQ water and PEG 400 (55,7:44,3) at 3 pH: 2, 7 and 9

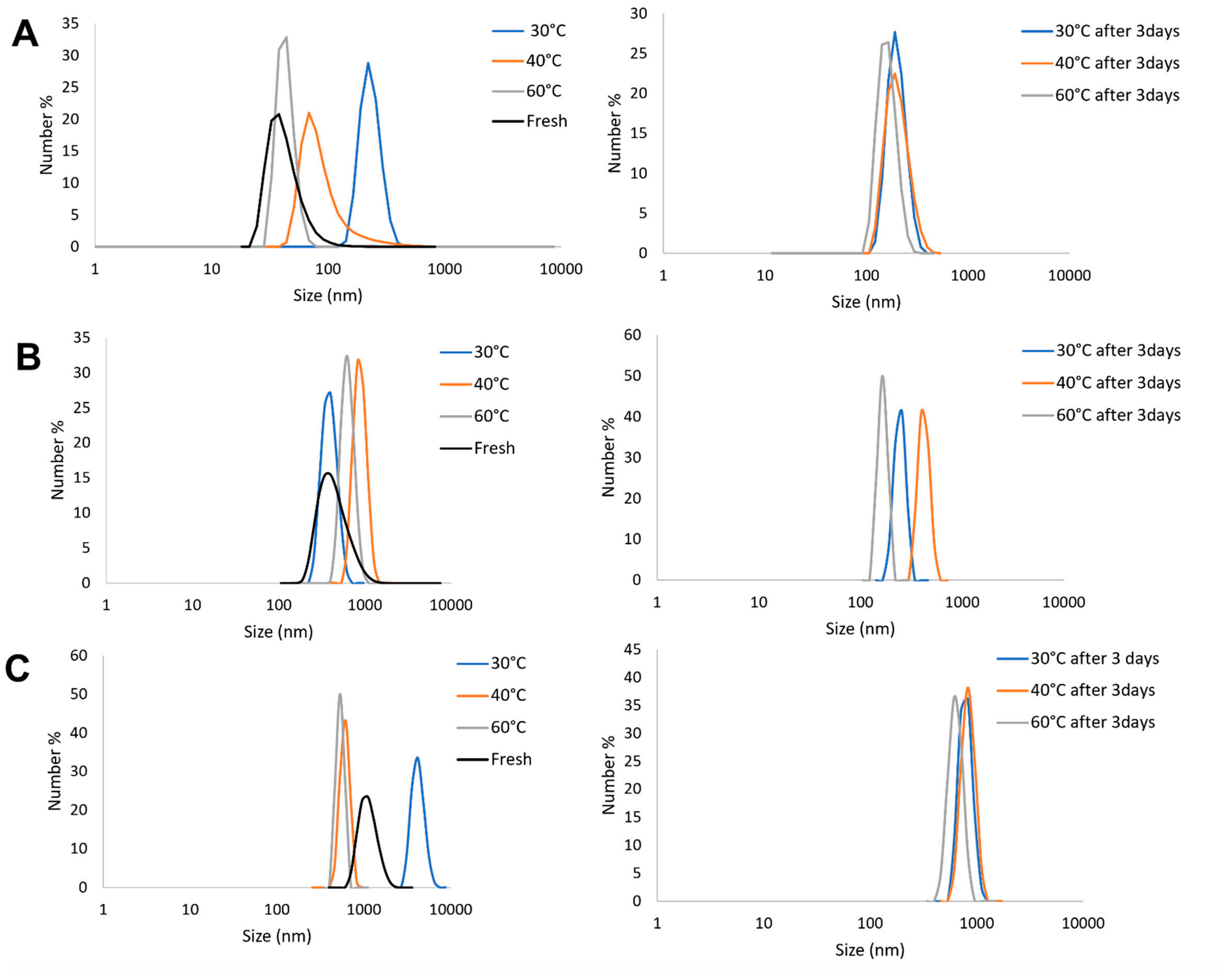

Figure S7. Size distribution curves of niosomes at 30°C, 40°C and 60°C fresh and after 3 days of storage. A) Niosomes with Rifamycin S encapsulated hydrated with pure MilliQ; B) Niosomes with Rifamycin S encapsulated hydrated with glycerol solution (60:40 v/v); C) Niosomes with Rifamycin S encapsulated hydrated with PEG 400 solution (44.7:55.3 v/v).

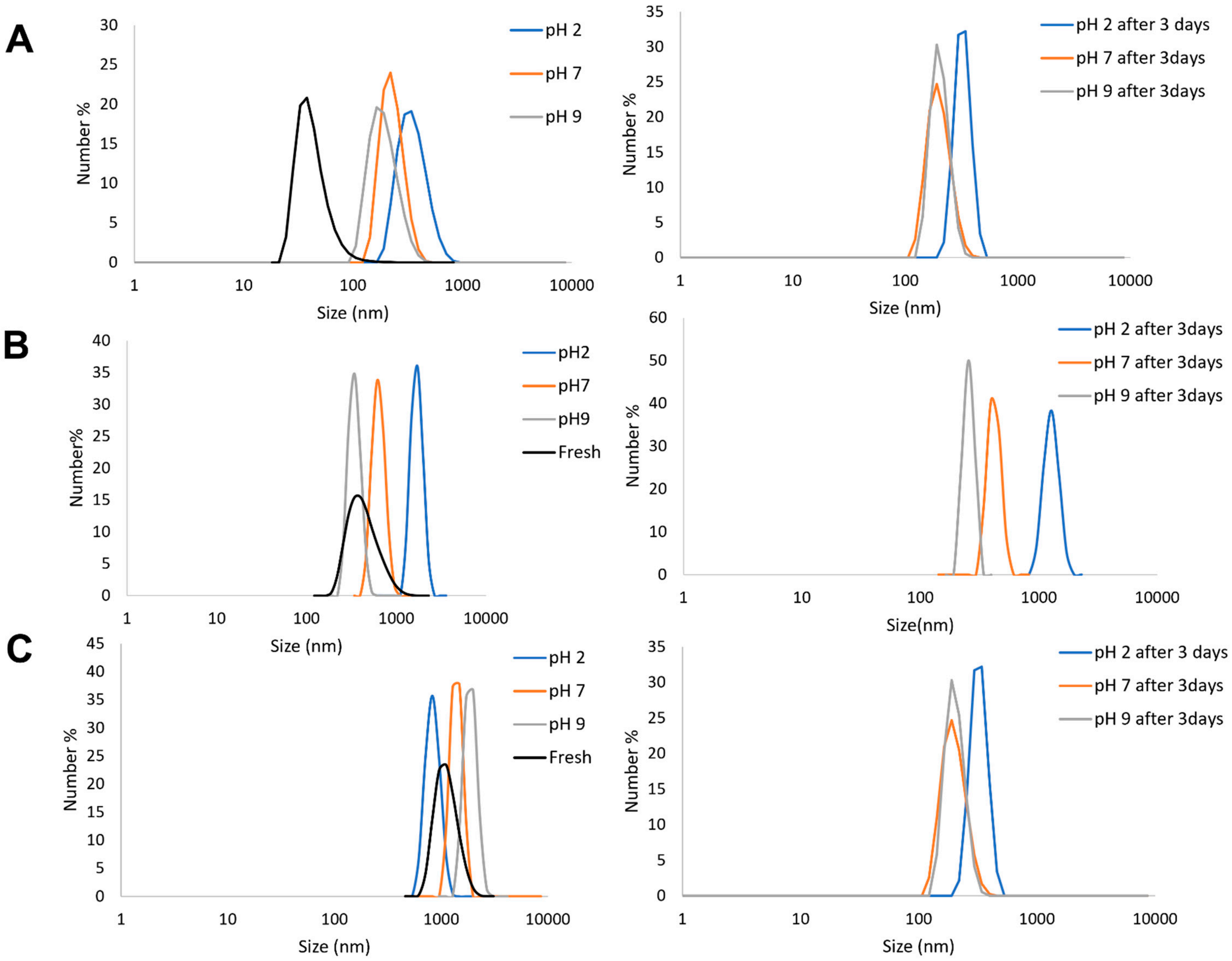

Figure S8. Distribution curves of niosomes at pH 2,7 and 9 in day 0 and day 3. A) Niosomes with rifamycin encapsulated in milliQ water as aqueous phase; B) Niosomes with ri-famycin encapsulated in mixture of milliQ water and glycerol (60:40 v/v); C) Niosomes with rifamycin encapsulated in mixture of milliQ water and PEG 400 (44.7:55.3 v/v).
